# Ionized calcium and ionized magnesium disturbances in dogs and cats with septic peritonitis

**DOI:** 10.3389/fvets.2025.1550701

**Published:** 2025-05-16

**Authors:** Chiara Debie, Laure Giberto, Stéphanie Noel, Dominique Paepe, Kris Gommeren

**Affiliations:** ^1^Small Animal Department, Faculty of Veterinary Medicine, Ghent University, Merelbeke, Belgium; ^2^Department of Clinical Sciences, FARAH, Faculty of Veterinary Medicine, University of Liège, Liège, Belgium

**Keywords:** ionized hypocalcemia, ionized hypermagnesemia, critical illness, calcium metabolism, septic abdomen, ionized hypomagnesemia

## Abstract

**Introduction:**

Hypocalcemia and magnesium disturbances are linked to vasoplegia, cardiac arrhythmias, gastrointestinal ileus, and coagulopathies. In human critical care patients, these imbalances are associated with higher mortality and longer hospital stay. Little is known about such associations in companion animals. Our study assessed the prevalence of ionized calcium (iCa) and ionized magnesium (iMg) disturbances in dogs and cats with septic peritonitis at presentation, and their association with administration of antiarrhythmics, vasopressors, prokinetics, and plasma, hospitalization duration and mortality.

**Methods:**

Medical records of patients with septic peritonitis from January 2018 to December 2023 were reviewed. Inclusion criteria were confirmed septic peritonitis and blood gas analysis with ionized calcium and magnesium values at admission. Data collected included signalment, diagnosis and cause, calcium and magnesium levels, administration of vasopressors, antiarrhythmics, prokinetics, and plasma, length of hospitalization, and survival. Comparisons were made using Chi-square, Fisher exact test, and ANOVA. Correlations were assessed with the Spearman coefficient.

**Results:**

Sixty-one dogs and 17 cats were included. Hypocalcemia (iCa < 1.25 mmol/L in dogs; <1.10 mmol/L in cats) was diagnosed in 51.7% of dogs and 23.5% of cats. Hypomagnesemia (iMg < 0.43 mmol/L in dogs; <0.47 mmol/L in cats) was found in 13.5% of dogs and 0% of cats, and hypermagnesemia (iMg > 0.6 mmol/L in dogs; >0.7 mmol/L in cats) in 15.4% of dogs and 60% of cats. Hypocalcemia and hypermagnesemia were significantly more common in dogs and cats, respectively. Ionized hypocalcemia was associated with plasma administration in dogs (*p* = 0.038). No significant correlation was found between iCa and iMg disturbances and length of hospitalization in dogs (respectively *p* = 0.62 and *p* = 0.62) or cats (respectively *p* = 0.62 and *p* = 0.27). The survival rate was 50%, with no association between iCa and iMg derangements and survival in dogs (*p* = 0.30 and *p* = 0.38 respectively), nor in cats (*p* = 0.29 and *p* = 0.29 respectively). A significant difference was observed in the mean iMg values between survivors (0.49 ± 0.09 mmol/L) and non-survivors (0.55 ± 0.11 mmol/L) in dogs (*p* = 0.042).

**Discussion:**

In this population with septic peritonitis, about half of the dogs and a quarter of the cats had ionized hypocalcemia. Hypo- and hypermagnesemia were rare in dogs, while hypermagnesemia was common in cats and hypomagnesemia was not seen in cats. Dogs and cats with hypocalcemia were more likely to receive plasma. Mean iMg values were lower in survived dogs than dogs that did not survive.

## 1 Introduction

Ionized hypocalcemia is a common electrolyte abnormality in critically ill people (1), with a prevalence described between 20 and 70% in septic patients (2, 3). The cause of ionized hypocalcemia in critically ill patients is likely multifactorial, with key pathways including parathyroid gland dysfunction (3), hypomagnesemia, acute renal failure (1–3), and calcitriol deficiency (3). Other contributing factors, such as chelation of calcium with lactate or free fatty acids (4), vitamin D deficiency (3), alkalosis (2), and acute pancreatitis (2), have also been reported. Calcium has important physiologic functions and hypocalcemia can lead to hypotension, systolic dysfunction, arrythmias, coagulopathies and functional gastro-intestinal ileus (3, 5–7). Moreover, hypocalcemia has been associated with increased mortality in critically ill people (1–3, 8).

Ionized hypocalcemia has been reported between 16 to 24% in critically ill dogs (9–11), and 59 to 93% in cats with septic peritonitis (12, 13). In critically ill dogs higher prevalences were observed with kidney disease, diabetes ketoacidosis, pancreatitis and sepsis (9). Ionized hypocalcemia in dogs has been correlated with longer hospitalization (9), and has been inconsistently associated with mortality (9–11). The study by Kellett-Gregory et al. is the only study that examined the clinical impact of ionized hypocalcemia. Their findings did not demonstrate an association between ionized hypocalcemia in cats and an increased prevalence of hypotension, coagulopathy, or arrhythmias during hospitalization (13).

Magnesium disorders have also been associated with hypotension/hypertension (14, 15), arrythmias (16–18), muscle spasms/weakness, refractory hypokalemia (19, 20), hypocalcemia (21), impaired insulin sensitivity (22, 23) or functional gastro-intestinal ileus in both human and veterinary medicine (24–27). Ionized magnesium alterations have been described in critically ill humans, with an incidence of 9.7–26% of ionized hypomagnesemia, and 15–23.6% of hypermagnesemia during hospitalization (8, 28, 29). Both hyper- and hypomagnesemia, whether present at admission or developed during hospitalization, have been associated with an increased mortality in critically ill humans (8, 28, 29).

Several studies on total serum magnesium levels in critically ill dogs found that 45–54% had hypomagnesemia and 8–13% had hypermagnesemia upon admission (11, 30). Another study investigated the prevalence of total magnesium disturbances in a specific cohort of hospitalized dogs with ionized hypocalcemia (31). This study reported a lower prevalence of total hypomagnesemia (22%) and total hypermagnesemia (3%) in this specific subgroup. Additionally, it found no correlation between ionized calcium (iCa) and total magnesium. Nevertheless, multiple studies have shown poor to moderate correlation between ionized magnesium and total magnesium, highlighting the limitations of using total magnesium as a reliable indicator of the magnesium status (28, 32–35). Moreover, in hospitalized and sick animals, the correlation between ionized magnesium and total magnesium tends to weaken (35). Studies investigating ionized magnesium levels in critically ill small animals are limited. One study examined the prevalence of ionized magnesium disturbances in critically ill dogs with parvoviral enteritis (34). In a study population of 72 dogs, a low prevalence of ionized hypomagnesemia (1.4%) was identified, while 6.9% of dogs had ionized hypermagnesemia. Another study of Murray et al. from 2023 investigated ionized magnesium disturbances in a more general population of hospitalized dogs. In this study, they identified a prevalence of 67% of ionized hypomagnesemia, and 4% of hypermagnesemia. In cats, total magnesium derangements were detected in 46% of cases (36). In both species, these derangements were associated with a longer hospital stay and higher mortality (30, 36). No study investigated the clinical impact of magnesium disturbances on the development of specific complications such as arrhythmias, hypotension or gastro-intestinal motility during hospitalization. Therapeutic value of magnesium supplementation in septic patients has not been established yet. Experimental studies in mice have demonstrated that magnesium supplementation mitigates the risk of developing sepsis (37), suggesting a potential therapeutic role for magnesium in critically ill patients during their hospitalization through its immunomodulatory effects.

The objectives of this study were to retrospectively evaluate the prevalence of ionized calcium and magnesium derangements at presentation in dogs and cats with septic peritonitis. Differences in the prevalence of calcium and magnesium derangements between dogs and cats were assessed. Additionally we investigated whether calcium and magnesium derangements at presentation were associated with increased morbidity and mortality. Our hypotheses were that ionized hypocalcemia, ionized hypermagnesemia and ionized hypomagnesemia were common in dogs and cats with septic peritonitis, and that such derangements were associated with increased vasopressor use, antiarrhythmic use, prokinetic use, plasma use, longer hospitalization and higher mortality.

## 2 Materials and methods

Medical records of dogs and cats presented to the emergency department of the University Veterinary Hospital of the University of Liège were searched to identify all dogs and cats with septic peritonitis between January 2018 and December 2023. Only dogs and cats with a confirmed diagnosis of septic peritonitis and with an available blood gas analysis including iCa and iMg values performed within the first 2 h upon presentation were included. The diagnosis of septic peritonitis was based on the presence of one of the following criteria: intracellular bacteria on cytology of peritoneal free fluid, bacterial growth on culture of peritoneal free fluid, or the identification of a pneumoperitoneum on abdominal ultrasound performed by a board certified imaging specialist in the absence of a history of a penetrating wound or abdominal surgery in the previous week, or identification of lesions compatible with a septic peritonitis during exploratory celiotomy (38, 39). Exclusion criteria were the administration of diuretics in the last 12 h before admission or blood products before the sample for blood gas analysis was taken.

Blood gas analysis was performed on commercial preheparinized syringes (Sarstedt Blood Gas Monovette®, Sarstedt AG & Co. KG, Nümbrecht, Germany) with a Calcium balanced lithium heparin solution. All samples were analyzed in an ion-selective electrode analyzer (Stat Profile Prime Plus® VET Critical Care Blood Gas Analyzer, Nova Biomedical, Waltham, Massachusetts, USA) within 15 min after collection. Reference intervals for ionized calcium were 1.25–1.5 mmol/L for dogs and 1.1–1.4 mmol/L for cats (40). Reference intervals for ionized magnesium were 0.43–0.6 mmol/L for dogs and 0.47–0.7 mmol/L for cats (27).

Medical records were reviewed for all included dogs and cats. Signalment, diagnosis and underlying cause of septic peritonitis, values of iCa and iMg upon admission, outcome, interventions during hospitalization and length of hospitalization were recorded. The administration of vasopressors, antiarrhythmics, prokinetics, and plasma transfusions during hospitalization were recorded, as crude markers of possible vasoplegia, cardiac arrhythmias, gastrointestinal stasis, and coagulopathies, respectively. Outcome was assessed at the time of discharge and categorized as survival or death, with the latter further classified into euthanasia and natural death. All euthanized dogs and cats were included in the prevalence and mortality analysis. However, for the morbidity analysis (association with therapeutic interventions), dogs and cats that were euthanized upon admission without undergoing hospitalization were excluded. Mean values of ionized calcium and ionized magnesium were analyzed and compared only for morbidity in dogs, as the small sample size in cats precluded statistical analysis.

### 2.1 Statistical analysis

Categorical variables were described using frequency tables (number and percent) and continuous variables were described using mean and standard deviation (±SD) or median and interquartile range (Q1–Q3) as appropriate. Normality of distributions was checked graphically. Comparison of categorical variables was done using Chi-square or Fisher exact test, ANOVA was used for continuous variables. Correlation was investigated using Spearman correlation coefficient. Results were considered statistically significant at 0.05 level (*p* < 0.05), no correction of multiple testing was applied. Analyses were conducted on the maximum number of observations, missing values were not replaced. Calculations were conducted using SAS (version 9.4) software (SAS version 9.4, SAS Institute Inc., Cary, North Carolina, USA). Boxplots were generated using R (Version 4.3.0) software (R Foundation for Statistical Computing, Vienna, Austria).

## 3 Results

**Figure 1:**
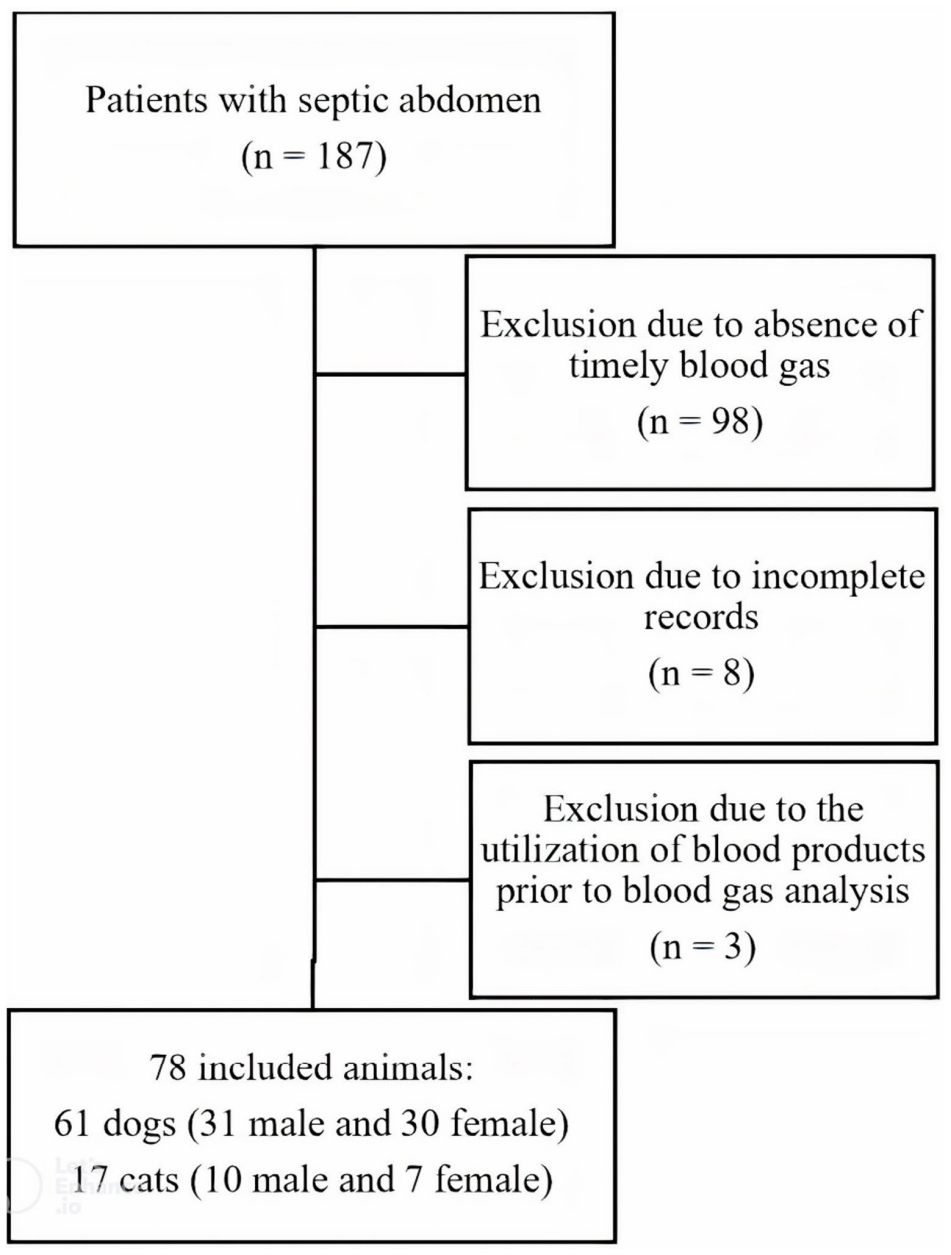
Flow chart representation of inclusion and exclusion process.

A total of 187 records of dogs and cats with a septic abdomen were identified, of which 109 were excluded. Exclusion was due to the absence of a timely performed blood gas (98), incomplete medical records (8) or the utilization of blood products prior to blood gas analysis (3). Details of exclusion can be found in Figure 1. Therefore, 61 dogs and 17 cats were included in the study. A total of 41 male dogs and cats were included (16 intact and 25 neutered), and 37 female (11 intact and 26 neutered). Mean age across all dogs and cats was 6.3 ± 3.7 years (6.5 ± 3.4 years in dogs; 5.5 ± 4.7 years in cats). Demographic data is represented in Table 1. There were 41 different dog breeds, with the most common ones crossbreeds (9), Belgian Malinois (3) and chihuahuas (3). There were 7 different cat breeds with domestic shorthair (9) being the most frequent. Underlying causes of septic peritonitis were gastrointestinal disruption in 47 dogs and cats (60.3%, 37 dogs and 9 cats), disruption of the urogenital tract in 9 (11.5%; 6 dogs and 3 cats), trauma in 4 (5.1%; 3 dogs and 1 cat), miscellaneous in 5 (6.4%; 4 dogs and 1 cat) and unknown in 13 cases (16.7%; 11 dogs and 2 cats).

**Table 1:** Demographic data and prevalence of iCa and iMg disturbances in dogs and cats with septic peritonitis.

**Parameters**	**All animals** **(*N* = 78)**	**Dogs** **(*N* = 61)**	**Cats** **(*N* = 17)**	***p*-value**
**Age (years)**	6.3 ± 3.7	6.5 ± 3.4	5.5 ± 4.7	
**Gender**				
Male	41	31	10	
Female	37	30	7	
**Ionized calcium**				
Mean values (mmol/L)		1.2 ± 0.11 (*N* = 60)	1.2 ± 0.09 (*N* = 17)	
Ionized hypocalcemia	35 (45.5%; *N* = 77)	31 (51.7%; *N* = 60)	4 (23.5%; *N* = 17)	***p* = 0.04**
Ionized hypercalcemia	0 (0%; *N* = 77)	0 (0%; *N* = 60)	0 (0%; *N* = 17)	*p* = 1.0
**Ionized magnesium**				
Mean values		0.52 ± 0.10 (*N* = 52)	0.78 ± 0.17 (*N* = 15)	
Ionized hypomagnesemia	7 (10.4%; *N* = 67)	7 (11.7%; *N* = 52)	0 (0%; *N* = 15)	*p* = 0.58
Ionized hypermagnesemia	17 (25.4%; *N*= 67)	8 (15.4%; *N* = 52)	9 (60%; *N* = 15)	***p* = 0.0032**

Comparisons of the prevalence of ionized calcium (iCa) and ionized magnesium (iMg) abnormalities between dogs and cats were performed using an ANOVA test. A significant difference was found between dogs and cats for ionized hypocalcemia (*p* = 0.04) and ionized hypermagnesemia (*p* = 0.0032).

### 3.1 Calcium and magnesium disturbances

**Figure 2:**
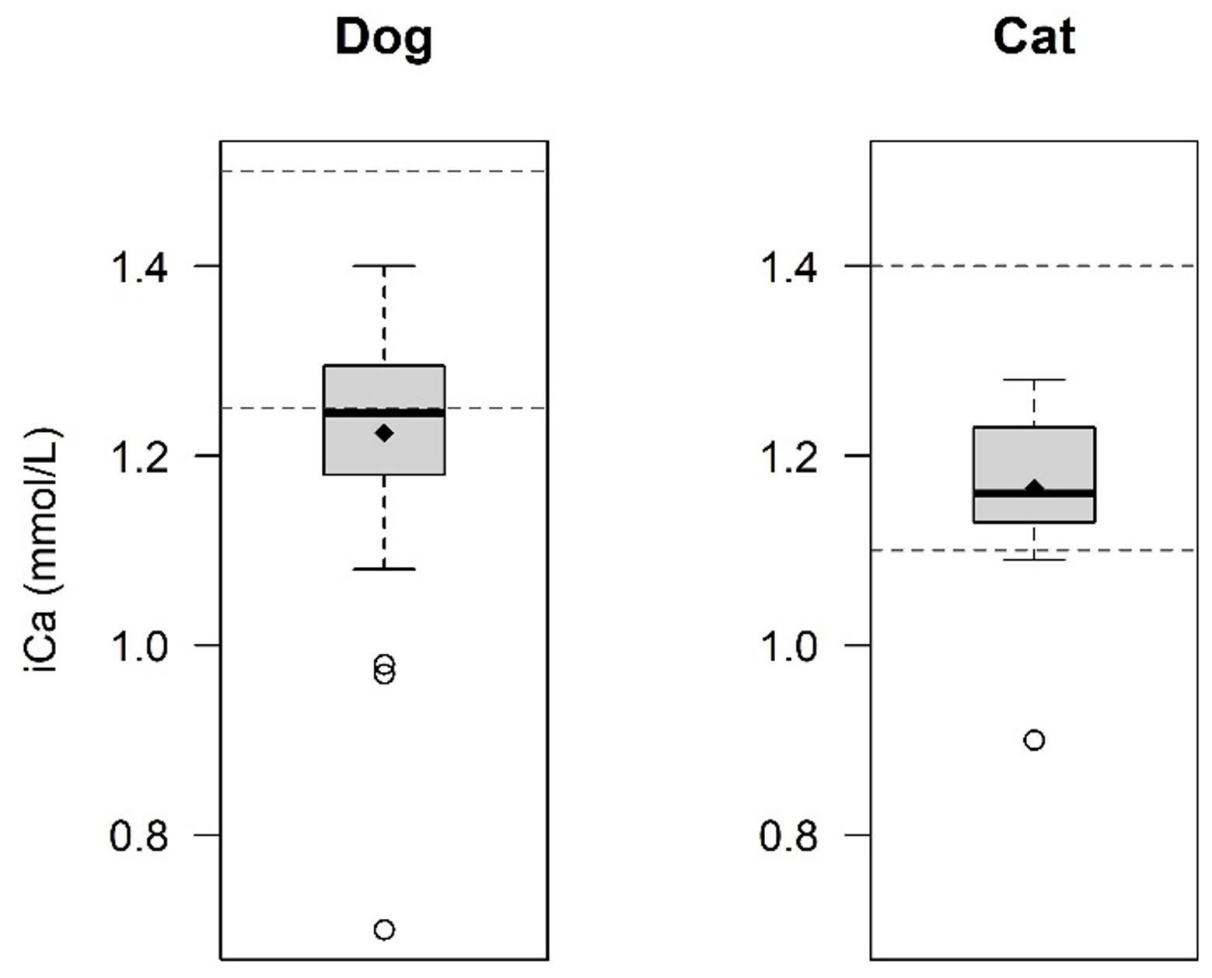
Comparison of ionized calcium (iCa) levels between dogs and cats. Ionized hypocalcaemia was significantly more frequent in dogs (51.7%) in comparison with cats (23.5%) (*p* = 0.040). The central line within each box represents the median, while the diamond represents the mean value. The box edges indicate the interquartile range (IQR; 25th-75th percentile). Whiskers extend to the smallest and largest values within 1.5x IQR, while points outside this range are considered outliers. The dashed lines indicate the normal reference range for dogs and cats.

**Figure 3:**
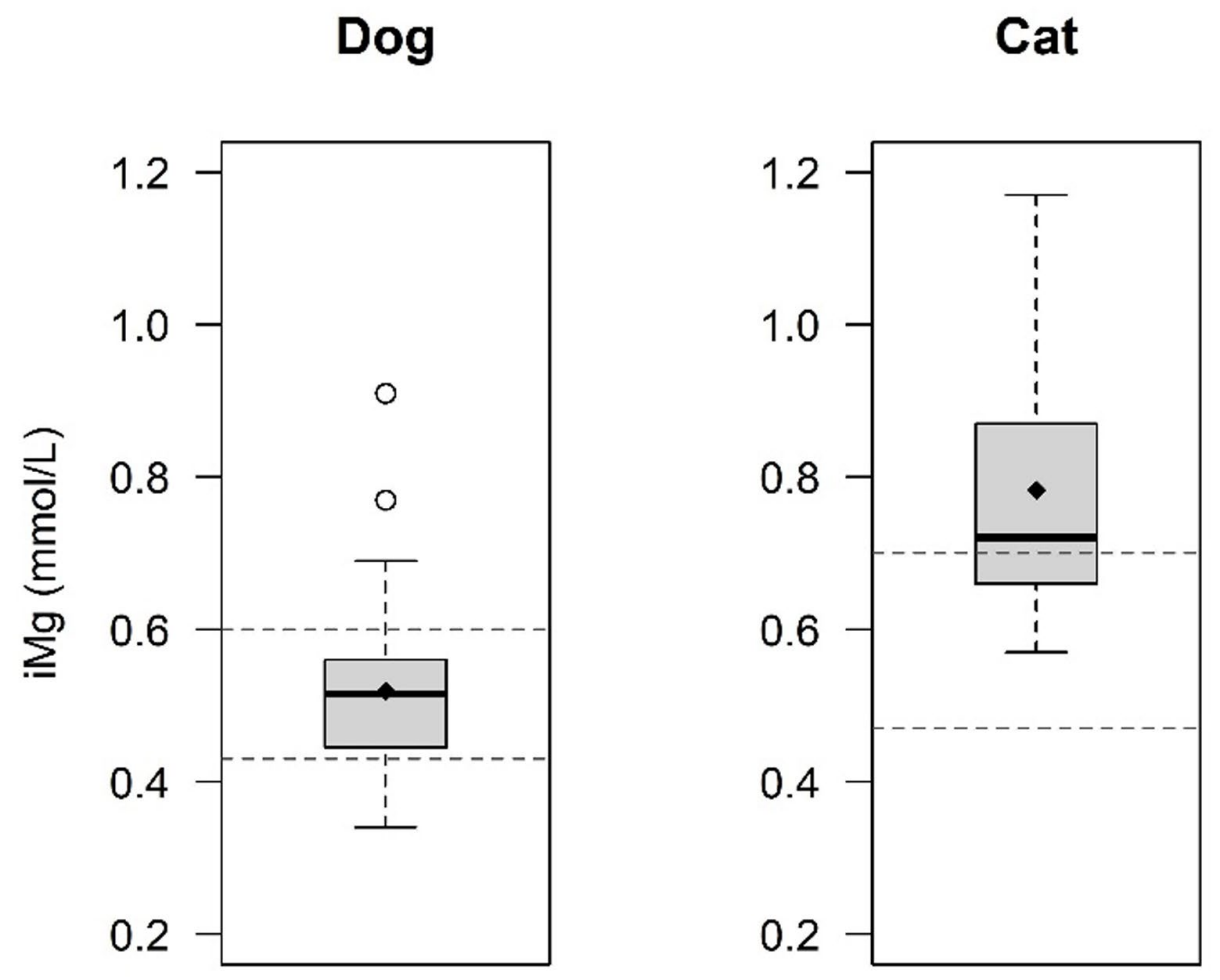
Comparison of ionized magnesium (iMg) levels between dogs and cats. Ionized hypermagnesemia is significantly more frequent in cats (60%) in comparison with dogs (13.3%) (*p* = 0.0032). The central line within each box represents the median, while the diamond represents the mean value. The box edges indicate the interquartile range (IQR; 25th-75th percentile). Whiskers extend to the smallest and largest values within 1.5x IQR, while points outside this range are considered outliers. The dashed lines indicate the normal reference range for dogs and cats.

Calcium measurements were not available in 1 dog and magnesium measurements were not available in 9 dogs and 2 cats. The mean (±SD) iCa level was 1.2 ± 0.11 mmol/L in dogs and 1.2 ± 0.09 mmol/L in cats, with 51.7% of dogs (31/60) and 23.5% of cats (4/17) had hypocalcemia while none had ionized hypercalcemia. Ionized hypocalcemia at presentation was observed more frequently in dogs compared to cats (*p* = 0.040) (Figure 2). The mean (±SD) iMg level was 0.52 ± 0.10 mmol/L in dogs and 0.78 ± 0.17 in cats. 13.5% of dogs (7/52) presented with ionized hypomagnesemia, while 15.4% of dogs (8/52) presented with hypermagnesemia. No cat (0%) was presented with ionized hypomagnesemia, while 60% of cats (9/15) were presented with ionized hypermagnesemia. Ionized hypermagnesemia at presentation was observed more frequently in cats compared to dogs (*p* = 0.0032) (Figure 3). The prevalence of calcium and magnesium disturbances is presented in Table 1.

### 3.2 Morbidity

The relationship between ionized calcium and ionized magnesium levels and the administration of vasopressors, antiarrhythmics, prokinetics, and plasma was analyzed in dogs, with the exclusion of dogs that were euthanized on admission. Due to the small sample size, the relationship between the presence of ionized hypocalcemia or ionized magnesium disturbances and the administration of vasopressors, antiarrhythmics, prokinetics and plasma could not be assessed in cats. In dogs, no statistically significant association was observed between the presence of hypocalcemia and the use of vasopressors (*p* = 0.32), antiarrhythmics (*p* = 0.68) or prokinetics (*p* = 0.72). A significant association was observed between ionized calcium levels and plasma administration during hospitalization in dogs. The prevalence of ionized hypocalcemia was higher in the plasma group, occurring in 73.3% of dogs compared to 41.2% in the group that did not receive plasma (*p* = 0.038). Results regarding the prevalence of ionized hypocalcemia in dogs and therapeutic interventions are represented in Table 2. The mean iCa concentration of dogs in the plasma group was 1.16 ± 0.16 mmol/L, compared to 1.25 ± 0.09 mmol/L in the group without plasma (*p* = 0.021). No significant difference could be found for the mean iCa concentration of dogs and the use of vasopressors (*p* = 0.24), antiarrhythmics (*p* = 0.52) and prokinetics (*p* = 0.60). Comparison of mean iCa values and administration of treatment during hospitalization in dogs is represented in Table 3.

**Table 2:** Association of disturbances in iCa and iMg with therapy during hospitalization in dogs with septic peritonitis.

**Treatment during hospitalization**	**Treatment given**	**Treatment not-given**	***p*-value**
**Hypocalcemia, *N* (%)**			
Vasopressors	17 (56.7)	8 (42.1)	*p* = 0.32
Antiarrhythmics	10 (47.6)	15 (53.6)	*p* = 0.68
Prokinetics	21 (52.5)	4 (44.4)	*p* = 0.72
Plasma	11 (73.3)	14 (41.2)	***p* = 0.038**
**Hypomagnesemia, *N* (%)**			
Vasopressors	4 (15.4)	3 (18.8)	*p* = 1.0
Antiarrhythmics	2 (11.8)	5 (20)	*p* = 0.67
Prokinetics	6 (17.1)	1 (14.3)	*p* = 1.0
Plasma	2 (16.7)	5 (16.7)	*p* = 1.0
**Hypermagnesemia, *N* (%)**			
Vasopressors	5 (19.2)	2 (12.5)	*p* = 0.69
Antiarrhythmics	2 (11.8)	5 (20)	*p* = 0.67
Prokinetics	6 (17.1)	1 (14.3)	*p* = 1.0
Plasma	3 (25)	4 (13.3)	*p* = 0.38

Comparisons were performed using a Chi-square or Fisher’s exact test to assess the association between the presence of an electrolyte disturbance (ionized hypocalcemia, ionized hypomagnesemia, or ionized hypermagnesemia) and the administration of specific treatments.

**Table 3:** Comparison of mean ionized calcium (iCa) values (mmol/L ± SD) in dogs and administration of treatment during hospitalization.

**Mean value iCa in mmol/L (N)**	**Treatment given**	**Treatment not-given**	***p*-value**
Vasopressors	1.21 ± 0.14 (30)	1.25 ± 0.09 (19)	*p* = 0.24
Antiarrhythmics	1.21 ± 0.13 (21)	1.23 ± 0.09 (28)	*p* = 0.52
Prokinetics	1.22 ± 0.13 (40)	1.24 ± 0.08 (9)	*p* = 0.60
Plasma	1.16 ± 0.16 (15)	1.25 ± 0.09 (34)	***p* = 0.021**

An ANOVA test was used for all comparisons. A significant difference was observed in mean iCa values between dogs that received plasma and those that did not (*p* = 0.021).

No significant association was found between the presence between hypo- or hypermagnesemia in dogs and the use of vasopressors (*p* = 1.0 and *p* = 0.69, respectively), antiarrhythmics (*p* = 0.67 and *p* = 0.67 respectively) prokinetics (*p* = 1.0 for both) or plasma (*p* = 1.0 and *p* = 0.38, respectively). Similarly, no association was found between the mean values of iMg in dogs and the use of vasopressors (*p* = 0.84), antiarrhythmics (*p* = 0.83), prokinetics (*p* = 0.25) and plasma (*p* = 0.86). Results regarding ionized hypomagnesemia or hypermagnesemia and therapeutic interventions are represented in Table 2. Comparison of mean iMg values and administration of treatment during hospitalization in dogs is represented in Table 4.

**Table 4:** Comparison of mean ionized magnesium (iMg) values (mmol/L ± SD) in dogs and administration of treatment during hospitalization.

**Mean value iMg in mmol/L ± SD (N)**	**Treatment given**	**Treatment not-given**	***p*-value**
Vasopressors	0.52 ± 0.1 (26)	0.51 ± 0.09 (16)	*p* = 0.84
Antiarrhythmics	0.51 ± 0.07 (17)	0.52 ± 0.11 (25)	*p* = 0.83
Prokinetics	0.51 ± 0.09 (35)	0.55 ± 0.12 (7)	*p* = 0.25
Plasma	0.51 ± 0.1 (12)	0.52 ± 0.1 (30)	*p* = 0.86

An ANOVA test was used for all comparisons. No significant differences were observed.

There was no significant correlation between iCa and iMg values and the length of hospitalization in dogs (*p* = 0.62 and *p* = 0.62 respectively). Similarly, no significant correlation was identified between iCa and iMg values and the length of hospitalization in cats (*p* = 0.62 and *p* = 0.27 respectively).

### 3.3 Mortality

**Figure 4:**
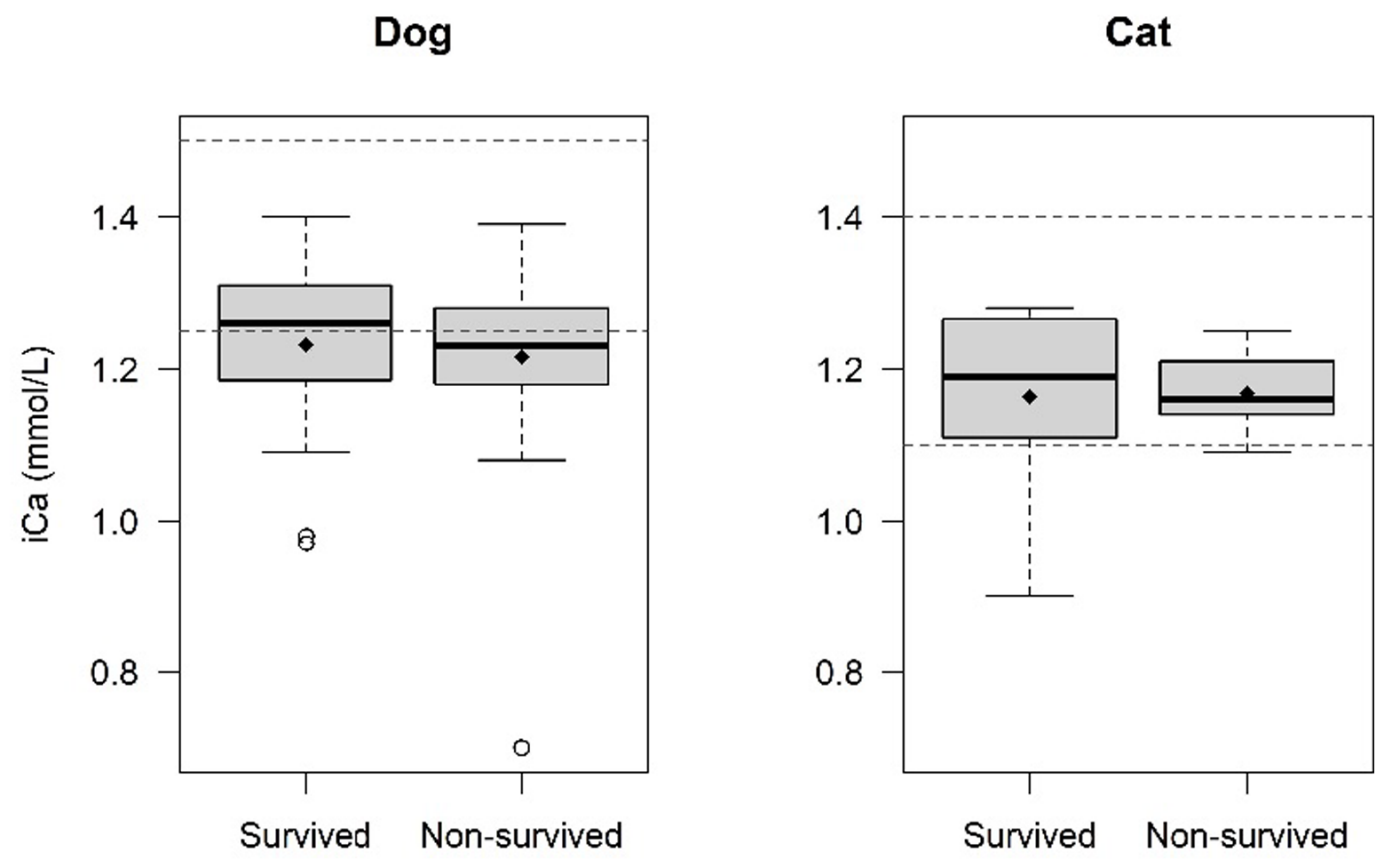
Comparison of ionized calcium (iCa) levels survivors and non-survivors. No statistical difference was found in mean iCa values between survivors and non-survivors in dogs (*p* = 0.59) and cats (*p* = 0.93). The central line within each box represents the median, while the diamond represents the mean value. The box edges indicate the interquartile range (IQR; 25th-75th percentile). Whiskers extend to the smallest and largest values within 1.5x IQR, while points outside this range are considered outliers. The dashed lines indicate the normal reference range for dogs and cats.

**Figure 5:**
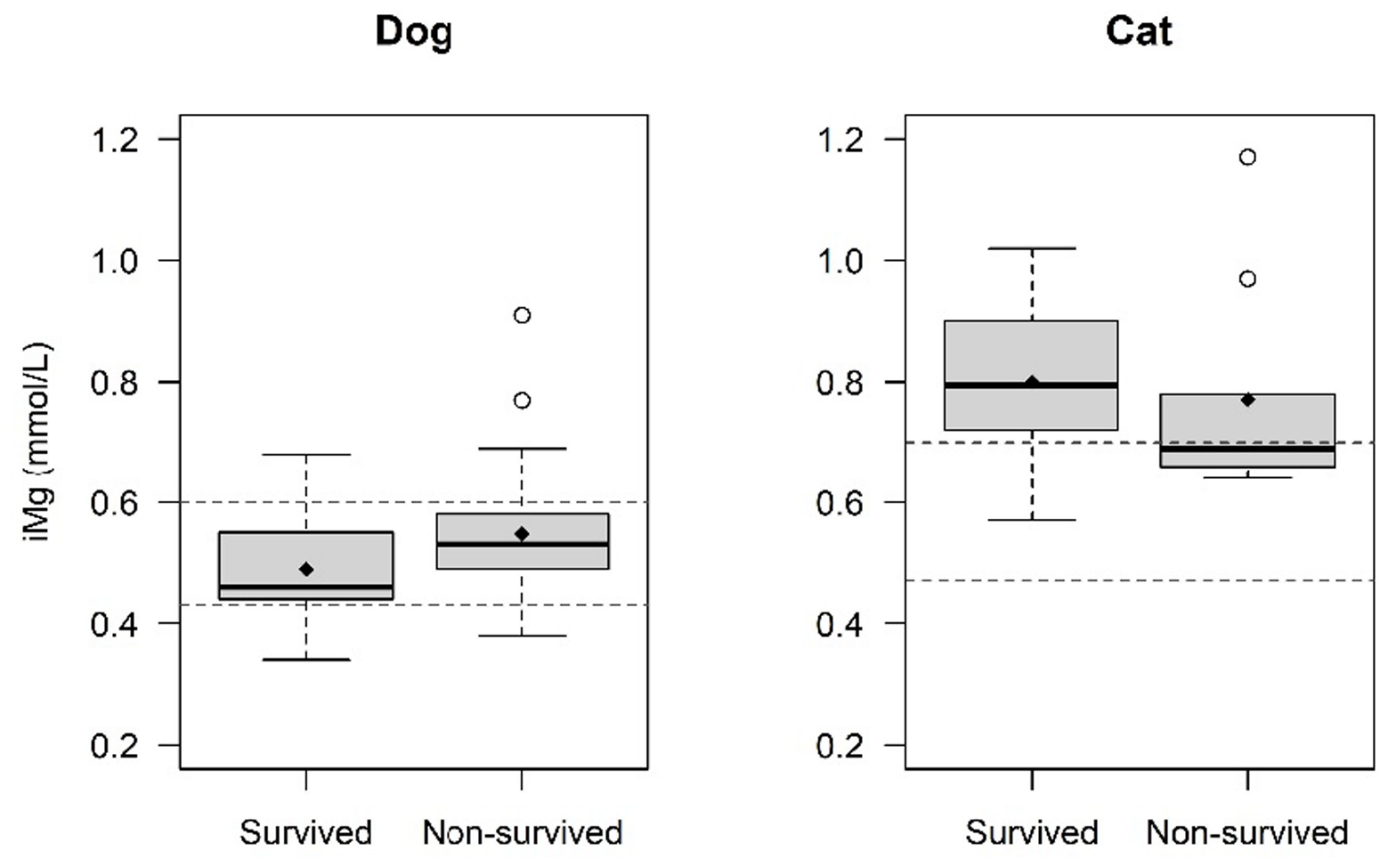
Comparison of ionized magnesium (iMg) levels between survivors and non-survivors. A statistically difference was found in mean iMg values between survivors and non-survivor in dogs. Mean iMg was lower in the survivors group (0.49 ± 0.09 mmol/L) compared to the non-survivors group (0.55 ± 0.11 mmol/L) (*p* = 0.042). No statistical difference was found in mean iMg values between survivors and non-survivors in cats (*p* = 0.76). The central line within each box represents the median, while the diamond represents the mean value. The box edges indicate the interquartile range (IQR; 25th-75th percentile). Whiskers extend to the smallest and largest values within 1.5x IQR, while points outside this range are considered outliers. The dashed lines indicate the normal reference range for dogs and cats.

The survival rate of the dogs and cats included in this study was 50%. Of the 39 dogs and cats that died, 27 were euthanized (34.6%, 15 of them at presentation prior to therapeutic intervention) and 12 died due to a cardiorespiratory arrest (15.4%). Of the dogs with ionized hypocalcemia, 45.2% survived (14/31), compared to 58.6 (17/29) in dogs with normocalcemia (*p* = 0.30). No statistically significant difference was observed in mean iCa values between survivors and non-survivors in dogs (*p* = 0.59) (Table 5 and Figure 4). Among dogs with ionized hypomagnesemia, 71.4% (5/7) survived, compared to 37.5 (3/8) in the hypermagnesemic group and 46% (17/37) in the normomagnesemic group (*p* = 0.38). A statistically significant difference was observed when comparing the mean iMg values between survivors and non-survivor in dogs. Mean iMg was lower in the survivors group (0.49 ± 0.09 mmol/L) compared to the non-survivors group (0.55 ± 0.11 mmol/L) (*p* = 0.042) (Table 5 and Figure 5).

**Table 5:** Comparison of mean ionized calcium (iCa) and ionized magnesium (iMg) values (mmol/L ± SD) in dogs and cats between survivors and non-survivors.

**Mean value in mmol/L ± SD (N)**	**Survivor**	**Non-survivor**	***p*-value**
**Dogs**			
iCa	1.2 ± 0.11 (31)	1.2 ± 0.12 (29)	*p* = 0.59
iMg	0.49 ± 0.09 (25)	0.55 ± 0.11 (27)	***p* = 0.04**
**Cats**			
iCa	1.2 ± 0.13 (8)	1.2 ± 0.05 (9)	*p* = 0.93
iMg	0.80 ± 0.16 (6)	0.77 ± 0.18 (9)	*p* = 0.76

An ANOVA test was used for all comparisons. A statistically significant diiference was found between mean iMg values in dogs between survivors and non-survivors (*p* = 0.04).

Of the cats with ionized hypocalcemia, 75% survived (3/4), compared to 38.5% (5/13) in the 13 cats with normocalcemia (*p* = 0.29). Among cats with ionized hypermagnesemia, 55.6% (5/9) survived, compared to 16.7% (1/6) in the normomagnesemic group (*p* = 0.29). No statistically significant difference was observed in mean iCa or iMg values between survivors and non-survivors in cats (*p* = 0.93 and *p* = 0.76 respectively) (Table 5 and Figures 4, 5).

## 4 Discussion

In this cohort, the prevalence of ionized hypocalcemia in dogs with septic peritonitis was found to be 51.7%, which is notably higher than the previously reported prevalence of 16–24% in critically ill dogs (9–11). In contrast, the prevalence of ionized hypocalcemia in this cohort of cats with septic peritonitis was 23.5%, which is lower than previously reported values of 59–89% of cats with septic peritonitis (12, 13). Ionized hypercalcemia was not observed in the present study. The previously published studies reports on the prevalence of ionized hypocalcemia in dogs focused on all dogs admitted to the intensive care unit (ICU) (9) or dogs with parvoviral enteritis (11). With the exception for the study of Mouton et al., which solely focused on the development of sepsis during hospitalization, these studies identified an association between ionized hypocalcemia and septic conditions. The higher prevalence in this cohort of dogs with septic peritonitis compared to an overall population of critically ill dogs was therefore expected. The prevalence of ionized hypomagnesemia and ionized hypermagnesemia was 13.5% and 15.4% for dogs and 0% and 60% for cats, respectively. As previously stated, studies for iMg are mostly lacking in veterinary medicine. The prevalence of derangements in total magnesium in critically ill dogs and cats was previously reported to be 53–67% and 46%, respectively (11, 30, 36). However, studies in human medicine and in dogs have shown only poor to moderate correlation between ionized magnesium and total magnesium (28, 32, 33). The underlying hypothesis is that total magnesium levels are influenced by serum albumin concentrations. Additionally, in critically ill patients, there may be a redistribution of magnesium from the extracellular to the intracellular compartment. This shift can result in decreased total magnesium levels while preserving normal ionized magnesium concentrations (32). Any such correlation between total and ionized magnesium has been reported to be even weaker in ill rather than in healthy dogs (35). Total magnesium and ionized magnesium cannot be used interchangeably, and direct comparison of the prevalence in our cohort with these previously published data studying total magnesium levels is therefore not possible. Compared to the study of Murray et al., the prevalence of ionized hypomagnesemia is lower, while the prevalence of ionized hypermagnesemia is higher in this cohort (35). Two main factors may explain these differences. First, our study sample is larger than the previous study, which could contribute to variations in prevalence. Secondly, there is a difference in disease severity between the two populations. Murry et al. included all patients admitted for hospitalization through an emergency department, whereas our study focuses exclusively on patients with septic peritonitis. In this cohort, patients are more prone to develop sepsis and secondary consequences, which may influence magnesium metabolism and excretion. Compared to the study by Mann et al., both ionized hypo- and hypermagnesemia are more prevalent in this study, despite comparable study sample sizes and disease severity (34). Additionally, both studies even used the same reference intervals for ionized magnesium. A difference in disease severity could have explained this discrepancy; however, no direct comparison between the two studies was possible in this regard.

### 4.1 Morbidity

This is one of the first studies in veterinary medicine that investigates the clinical impact of disturbances in ionized calcium or ionized magnesium in dogs and cats with a septic peritonitis. In this study, a significant association was identified between ionized hypocalcemia and plasma administration during hospitalization in dogs. Plasma is commonly administered for managing hypocoagulability, and thus was used as a crude marker to explore the relationship between iCa levels and coagulopathies. Calcium (Ca^2+^) plays a critical role in multiple stages of the coagulation cascade (41), and hypocalcemia is associated with impaired coagulation. Plasma is also used for oncotic support in animals with hypoalbuminemia. Although hypoalbuminemia is associated with decreased total calcium levels [roughly 40% of circulating calcium is protein bound, it does not affect ionized calcium (42)].

Although both ionized calcium (iCa) and ionized magnesium (iMg) abnormalities can contribute to hypotension due to vasoplegia, cardiac arrythmias and gastrointestinal ileus, no significant association was observed between electrolyte derangements and the use of antiarrhythmics, prokinetics or vasopressors. However, we did not study whether patients demonstrated hyperemic mucous membranes or developed hypotension due to vasoplegia, nor did we assess motility via monitoring of by abdominal POCUS (43) or recording of gastric residual volume through a nasogastric feeding tube. Neither an ECG interpretation was included in this study. The administration of antiarrhythmics, vasopressors or prokinetics again served as a crude marker in an attempt to record associations between calcium and magnesium derangements and these complications.

No significant correlation was observed between iCa or iMg levels and the length of hospitalization in this study. This finding contrasts with a previous study by Holowaychuk et al., which reported a significantly longer hospitalization duration in dogs with ionized hypocalcemia (9). Similarly, a human study in critically ill paediatric patients found a significant correlation between hypocalcemia and the length of hospitalization (8). In contrast, our results align with those of Kellett-Gregory et al., who similarly found no association between iCa and iMg derangements in a disease group that is similar to ours (13). In the latter study, the authors hypothesized that variability in the administration of fluids or blood products prior to iCa measurement could have influenced results, but the design of our study appears to suggest differently.

Prior research in veterinary (36) and human medicine (8) has identified associations between iMg imbalances and hospitalization duration. However, those studies assessed iMg fluctuations throughout the entire hospitalization period. As previously stated, the evaluation of iCa and iMg during hospitalization was outside the scope of our study.

### 4.2 Mortality

The survival rate until discharge of the dogs and cats in this study was 50%. This is lower than or comparable to survival rates of previous studies (34.4–85%) on septic peritonitis in companion animals (13, 44–58). That said, our study included all dogs diagnosed with septic peritonitis and an available blood gas. As a consequence, the survival rate is potentially negatively impacted by the significant amount of dogs and cats euthanized after diagnosis without therapeutic intervention. Although the inclusion criteria for septic peritonitis were broad and could include dogs and cats with only a positive culture growth, more than 50% of treated dogs and cats required more than one vasopressor during hospitalization, indicating systemic involvement in our cohort. The current study did not identify an association between survival and the prevalence of iCa and iMg derangements, similarly to the papers from Holowaychuk et al. and Kellet-Gregory et al. This is in contrast with human and other veterinary medicine papers (1–3, 8, 10, 29, 30, 36, 59). In dogs, survivors had a significantly lower mean iMg concentration, although mean iMg values in both groups were within the reference range used in this study. In human studies, several hypotheses have been proposed to explain the association between elevated iMg and increased mortality. Ionized magnesium may serve as a marker of cellular damage, may indicate decreased renal perfusion, or reflect alterations due to acid–base imbalances (8, 28, 60). That said, our study was performed on a small sample, and values were only assessed upon presentation, and the clinical relevance of this observation therefore remains uncertain. Other studies additionally demonstrated an association between iCa and iMg levels during hospitalization and survival. A human paper showed higher mortality in critically ill patients that developed hypermagnesemia during hospitalization (28), whereas a study on ionized hypocalcemia in cats with septic peritonitis identified decreased survival in cats in which calcium levels failed to normalize (13). Assessing the evolution of iCa and iMg levels throughout hospitalization would have been insightful, but this was beyond the scope of the current study. A study on the evolution of these electrolytes during hospitalization would also require a larger sample size to account for the loss of patients during hospitalization and the large number of confounding factors. During the treatment of septic peritonitis numerous factors can indeed influence calcium and magnesium levels. To name a few, the administration of citrated blood products (61, 62), colloids (63) or 0.9% saline infusion can reduce iCa and iMg levels due to dilution effects or the calcium-binding properties of citrate (5, 24, 40, 64, 65).

### 4.3 Limitations

This study has several limitations. The reference ranges used in this study are those established by the authors institute, but were based on reference ranges in literature (40, 66). For the analyzer used in the study, the manufacturer did not establish internal reference ranges, and, to the author’s knowledge, no study has yet defined reference ranges specifically for this analyzer. The lack of an internal reference range may explain differences in prevalence rates compared to other studies. However, reference ranges in different sources based on different analyzers only show mild differences (5, 67, 68).

Due to the retrospective design of this study, blood samples were collected by multiple clinicians without standardized protocols for syringe filling, potentially introducing dilutional errors with heparin that could affect the measurements of ionized calcium (iCa) and ionized magnesium (iMg) (5, 27, 40, 66, 69). The measurement of ionized calcium (iCa) and ionized magnesium (iMg) can indeed be influenced by various factors. Proper sample collection and handling are critical, as chelation of Ca^2+^ and Mg^2+^ can occur in the presence of heparin or lactate (5, 27, 40, 66). Additionally, the presence of air bubbles in samples can affect iCa levels, as the alkalinization caused by gas exchange promotes increased protein binding of calcium, thereby reducing the ionized fraction of calcium (5, 66, 70). Our retrospective design does not allow us to exclude any of these effects on our samples.

Several comorbidities may influence iCa and iMg levels. Conditions such as trauma (71), neoplasia (72), renal failure (17, 72, 73), hyperthyroidism (64), pancreatitis (24, 74–76), hypoadrenocorticism (72), and diabetes mellitus (24, 77, 78), are known to affect iCa and iMg values. Similarly, medications such as furosemide (17, 64) and corticosteroids (5, 79) impact iCa and iMg levels. Additionally, several isotonic crystalloids, such as Lactated Ringer’s solution or Plasmalyte, contain small concentrations of calcium or magnesium (80). Dogs and cats that received these solutions from the referring veterinarian prior to arrival were not excluded from the study. In our study, several dogs and cats were affected by such conditions, or did receive treatments known to influence iCa and iMg levels. Three dogs and 1 cat had septic peritonitis secondary to trauma, 4 received long-term corticosteroid treatment, 1 received long-term furosemide therapy, and similarly there was a single patient suffering from chronic kidney disease, acute renal failure, apocrine gland anal sac adenocarcinoma, or hyperthyroidism. Previous observational studies in both human and veterinary medicine (2, 8–10, 13, 28–30, 81) did not exclude patients with these comorbidities.

An additional limitation of this study is the small sample size. The limited overall sample size may have influenced results, as some borderline findings could have reached significance with a larger sample. Caution should therefore be warranted when interpreting the potential influence of comorbidities. As there is an indication that dogs and cats might have a difference in the metabolism of calcium (82) and magnesium (83), it was decided to investigate dogs and cats as separate groups. Due to the limited sample size in cats, morbidity analysis could not be conducted. Larger prospective studies are warranted to avoid any type II error.

The possible associations between iCa and iMg disturbances and arrhythmias, vasodilatory shock, gastrointestinal ileus, and coagulation disorders were not directly assessed as our medical records did not allow to diligently track this information. Rather we recorded the use of vasopressors, anti-arrhythmics, prokinetics, and plasma transfusions and investigated their association with iCa and iMg derangements. The authors acknowledge the strong limitation of such analyses, as treatments may have been motivated by other factors, and inversely a lack of treatment does not imply an absence of the condition. Any identified association or lack thereof should therefore be confirmed in further prospective studies in which precise monitoring for such conditions seems warranted.

In conclusion, ionized hypocalcemia was significantly more common in dogs than cats with septic peritonitis and associated with an increased use of plasma during hospitalization. The prevalence of hypo- and hypermagnesemia in dogs was low, no correlation of magnesium disturbances had a significant clinical impact. Hypermagnesemia, although still rare in cats, was significantly more common than in dogs, whereas hypomagnesemia was not detected. The clinical significance of derangements at presentation in our study population seemed limited, without impact on survival to discharge.

## Data Availability

The raw data supporting the conclusions of this article will be made available by the authors, without undue reservation.
